# Commutability of Cytomegalovirus WHO International Standard in Different Matrices

**DOI:** 10.1128/JCM.03292-15

**Published:** 2016-05-23

**Authors:** Sara Jones, Erika M. Webb, Catherine P. Barry, Won S. Choi, Klara B. Abravaya, George J. Schneider, Shiaolan Y. Ho

**Affiliations:** Abbott Molecular, Des Plaines, Illinois, USA; Boston Children's Hospital

## Abstract

Commutability of quantitative standards allows patient results to be compared across molecular diagnostic methods and laboratories. This is critical to establishing quantitative thresholds for use in clinical decision-making. A matrix effect associated with the 1st cytomegalovirus (CMV) WHO international standard (IS) was identified using the Abbott RealTime CMV assay. A commutability study was performed to compare the CMV WHO IS and patient specimens diluted in plasma and whole blood. Patient specimens showed similar CMV DNA quantitation values regardless of the diluent or extraction procedure used. The CMV WHO IS, on the other hand, exhibited a matrix effect. The CMV concentration reported for the WHO IS diluted in plasma was within the 95% prediction interval established with patient samples. In contrast, the reported DNA concentration of the CMV WHO IS diluted in whole blood was reduced approximately 0.4 log copies/ml, and values fell outside the 95% prediction interval. Calibrating the assay by using the CMV WHO IS diluted in whole blood would introduce a bias for CMV whole-blood quantitation; samples would be reported as having higher measured concentrations, by approximately 0.4 log IU/ml. Based on the commutability study with patient samples, the RealTime CMV assay was standardized based on the CMV WHO IS diluted in plasma. A revision of the instructions for use of the CMV WHO IS should be considered to alert users of the potential impact from the diluent matrix. The identification of a matrix effect with the CMV WHO IS underscores the importance of assessing commutability of the IS in order to achieve consistent results across methods.

## INTRODUCTION

Management of human cytomegalovirus (HCMV) is an important aspect of treatment for transplant patients. Historically, the treatment options have been prophylactic treatment without regard to CMV status or preemptive treatment of patients whose level of CMV infection exceeds a predetermined threshold (1–4). CMV viral load thresholds have been established in transplant centers around the world. However, until recently, it was not possible to determine the relationships between measurements made from different tests because there was no common reference material. The 1st WHO International Standard for Human Cytomegalovirus for Nucleic Acid Amplification Techniques (CMV WHO IS; NIBSC code 09/162) and Standard Reference Material (SRM) 2366 Cytomegalovirus for DNA Measurements, from the National Institute of Standards and Technology (NIST), are tools that can be used to unify the reporting of CMV viral loads (5, 6). Determining the mathematical relationship between the concentration of the standard(s) and results reported in each assay makes it possible to standardize patient results from different assays.

A goal of laboratory medicine is that results for patient samples be comparable independent of the medical laboratories or methods which produce the results. In order to use a reference to standardize results among different methods, the material must be commutable, i.e., results obtained from measurement of the standard must reflect the measurement of the same analyte in patient samples (7–11). Standardization with a commutable reference ensures that the results for clinical samples assayed by different measurement procedures have numerical values that are equivalent, irrespective of the clinical method used for the measurement. Commutability was first used to describe the ability of an enzyme reference or control material to have interassay properties comparable to the properties demonstrated by authentic clinical samples when measured by more than one analytical method (7). The definition has been expanded to an equivalence of the mathematical relationships between the results of different measurement procedures for a reference material and for representative samples from healthy and diseased individuals (8–11).

For methods measuring CMV viral load, which can be performed with plasma or whole-blood (WB) patient specimens, depending on the testing laboratory, proper standardization to the CMV WHO IS both within and across matrices is an important consideration. Here we report the identification of a matrix effect associated with the CMV WHO IS, a commutability study to further characterize the effect, and the mitigation adopted in standardizing the Abbott RealTime CMV assay to address the uncovered bias.

## MATERIALS AND METHODS

### CMV standards.

The 1st WHO International Standard for Human Cytomegalovirus for Nucleic Acid Amplification Techniques (NIBSC code 09/162) comprises a whole-virus preparation of HCMV strain Merlin in Tris-HCl buffer with human serum albumin. It has been lyophilized in 1-ml aliquots. The lyophilized WHO IS was stored at −20°C prior to use. The WHO IS was reconstituted according to the manufacturer's instructions in 1 ml of nuclease-free water, to a nominal concentration of 5 × 10^6^ international units (IU)/ml. The reconstituted WHO IS was diluted in commercially available normal human EDTA-plasma, normal human EDTA-WB, or buffer (Tris-EDTA [TE] or TE with salmon testis DNA [Sigma-Aldrich] as a carrier). The normal human EDTA-plasma was purchased as a pool. The normal human WB was a pool of 4 units. Both plasma and WB pools were purchased from ProMedDx and were prescreened for the absence of CMV DNA by using the Abbott RealTime CMV assay. Dilutions were tested on the same day they were prepared or stored at −70°C for 7 days and then tested.

### SRM 2366.

The cytomegalovirus for DNA measurements from the National Institute of Standards and Technology (NIST) is purified DNA from the Towne strain of CMV in a bacterial artificial chromosome (Towne_Δ147_ BAC). SRM 2366 contains the entire CMV genome, except for the IRS1, US1-15, and UL147 regions (6). The material was quantified via digital PCR and was assigned a concentration reported in DNA copies per microliter. The NIST SRM was stored at 2 to 8°C prior to use. For the testing reported in Fig. 5, NIST SRM components A, B, and C were diluted 1:1,000 in pooled normal EDTA-plasma or -WB prescreened for the absence of CMV DNA. For the testing reported in Table 2, NIST SRM component C was diluted 1:2,000 in pooled normal EDTA-plasma, EDTA-WB, or buffer (TE or TE with carrier DNA). Dilutions were tested on the same day they were prepared.

### Patient samples.

CMV DNA-positive plasma specimens were leftover and deidentified samples from routine clinical testing obtained in accordance with applicable law and regulations. The samples were stored frozen at −70°C and thawed at the time of dilution. For the testing reported in Fig. 2 to 4, 20 CMV-positive plasma specimens were diluted 1:50 in pooled normal human EDTA-plasma and -WB prescreened for the absence of CMV DNA. Patient sample dilutions were tested on the day they were prepared.

### Testing procedures.

Dilutions of the standards and patient samples were extracted and amplified using the Abbott RealTime CMV assay on an Abbott *m*2000 system.

### Abbott RealTime CMV assay. (i) Assay procedures.

The Abbott RealTime CMV assay is carried out on an Abbott *m*2000 system. It has two automated test procedures, for processing plasma and whole-blood specimens. Sample preparation and PCR assembly are performed on an Abbott *m*2000sp instrument. Real-time PCR amplification and detection and result reporting are performed on an Abbott *m*2000rt instrument. The test procedures for plasma and whole blood share the same reagents for sample preparation, PCR amplification, and detection but differ in their sample input and elution volumes. DNA is extracted from 0.5 ml of a plasma sample and eluted in a 70-μl eluate or from 0.3 ml of a whole-blood sample and eluted in a 110-μl eluate. Plasma samples can be processed by either extraction procedure, but WB samples can be extracted only by the WB extraction procedure.

For both procedures, the total PCR volume is 60 μl (35 μl eluate and 25 μl master mix). An internal control (IC) is mixed into the lysis reagent before the initiation of sample preparation and is added to each specimen, calibrator, and control as a control for extraction efficiency and to monitor PCR inhibition.

### (ii) Targets.

The Abbott RealTime CMV assay amplifies two targets, within the UL34 and UL80.5 genes. The target regions were chosen for their conservation in human CMV (data not shown). The redundancy in target amplification is designed to provide robust, accurate, and sensitive quantitation of CMV DNA. The primer and probe binding regions used for the Abbott RealTime CMV assay are 100% identical to the Merlin and Towne strain CMV sequences represented in the CMV WHO IS and the NIST SRM 2366, respectively (data not shown).

The RealTime CMV assay also amplifies a noncompetitive IC derived from the hydroxypyruvate reductase gene from the pumpkin plant *Cucurbita pepo*. The CMV and IC probes are single-stranded DNA oligonucleotides modified with fluorescent and quenching moieties. The two CMV probes are labeled with the same fluorophore, and the IC probe is labeled with a different fluorophore. Signals for CMV and IC are detected simultaneously and distinguished.

### (iii) Assay calibration.

The RealTime CMV assay utilizes external calibration. The high and low calibrators are formulated as a linearized plasmid containing dual CMV targets (UL34 and UL80.5) in a buffered solution with carrier DNA. The DNA copy number is determined from the absorbance at 260 nm with purified plasmid DNA and is used to standardize the assay. The same calibrators are used for both plasma and WB extraction procedures. Calibrators are part of the test procedures and are processed through sample preparation. Representation of the dual CMV targets in calibration and the required processing of calibrators through sample preparation ensure proper quantitation of CMV DNA from both plasma and whole blood. The assay was optimized for highly sensitive quantitation of CMV DNA from both plasma and whole-blood matrices (data not shown). The claimed quantitation range is 20 copies/ml to 100 million copies/ml (1.30 to 8 log copies/ml) for plasma and 40 copies/ml to 100 million copies/ml (1.60 to 8 log copies/ml) for whole blood. The analytical lower limit of quantification (LLoQ) values reflect equivalent quantitation for the plasma and WB procedures after correcting for the approximately 2-fold difference in sample input and elution volumes.

### (iv) Assay controls.

Negative and positive controls are required for each run to verify that the procedure is performed correctly. The negative control is a buffered solution with salmon testis carrier DNA. The positive control is formulated with inactivated CMV strain AD169 in plasma.

### (v) Assay standardization to the 1st WHO IS.

To achieve assay standardization, a conversion factor was determined that establishes the mathematical relationship between the number of DNA copies used for the RealTime CMV assay and the number of international units. Briefly, the WHO IS reconstituted in water was diluted to concentrations of 5.02 log IU/ml and 3.52 log IU/ml in pooled plasma. The dilutions of the WHO standard were tested against the Abbott RealTime CMV primary calibrators. Twenty to 24 total replicates of each WHO IS dilution and each primary calibrator were tested across 2 runs. The median concentrations (in log copies per milliliter [log copies/ml]) for both WHO IS dilutions were calculated. The conversion factor for log copies/ml to log IU/ml was calculated as the difference between the stated concentration (5.02 log IU/ml) and the calculated median concentration (in log copies/ml) and was determined to be log copies/ml + 0.19 = log IU/ml. The conversion factor for copies/ml to IU/ml was calculated by dividing the stated concentration of the 5.02-log IU/ml dilution (104,967 IU/ml) by the median concentration (in copies/ml) and was determined to be copies/ml × 1.56 = IU/ml. The conversion factor between numbers of international units and DNA copies was verified by applying it to the 3.52-log IU/ml dilution of the WHO standard. Colinearity for the WHO standard was demonstrated throughout the concentration range of 50 IU/ml to 500,000 IU/ml (data not shown).

### Matrix effect study design and analysis.

The study to assess matrix effects was performed per Clinical and Laboratory Standards Institute (CLSI) guidance document EP14-A2, using patient samples as the standard of comparison (12). Twenty plasma specimens from CMV-positive patients, diluted 1:50 in pooled EDTA-plasma and -WB, were tested as single replicates in 3 runs, one on each of 3 *m*2000 instruments, for a total of 3 replicates per patient sample in each matrix. Due to constraints on the number of samples that can be handled at once, the patient sample dilutions were tested in groups of 10 on 2 days. Single replicates of the diluted WHO IS were run as samples on both days, for a total of 6 replicates in each matrix. All runs used Abbott RealTime CMV calibrators.

NIST SRM components A, B, and C were diluted 1:1,000 in pooled normal EDTA-plasma or -WB prescreened for the absence of CMV DNA and were tested as single replicates in 3 runs with the plasma procedure and 3 runs with the WB procedure, on each of 3 *m*2000 instruments, for a total of 3 replicates per component in each matrix. The NIST SRM dilutions were not run on the same day as the patient samples, but dilutions of 3 of the patient samples were included as controls in the NIST SRM runs.

Regression analysis was performed according to CLSI guidance document EP14-A2, using Microsoft Excel. Results for the different diluent matrices and different testing procedures were expressed in numbers of DNA log copies/ml and evaluated. Regression was performed using the means for patient specimen dilutions, and the two-tailed 95% prediction interval was calculated. Results for the dilutions of the CMV WHO IS and the NIST SRM were evaluated relative to the prediction interval.

The performance of the CMV WHO IS relative to patient samples was also determined using the Bland-Altman method (13, 14). The difference between conditions was plotted relative to the average.

## RESULTS

### Pilot study to assess CMV WHO IS diluted in plasma and whole blood.

The instructions for use provided with the 1st CMV WHO IS state that the material should be reconstituted to a nominal concentration of 5 × 10^6^ IU/ml in 1 ml of deionized nuclease-free water. Following reconstitution, the IS should be diluted in the matrix appropriate to the material being calibrated and should be extracted prior to CMV DNA measurement. Plasma and whole blood are the two common specimen types used in CMV patient management, and the Abbott RealTime CMV assay can be used to test either. The RealTime CMV assay has two automated test procedures: one for processing plasma specimens and the other for processing WB specimens. Plasma and WB were therefore deemed to be appropriate for diluting the reconstituted CMV WHO IS for testing.

**FIG 1:**
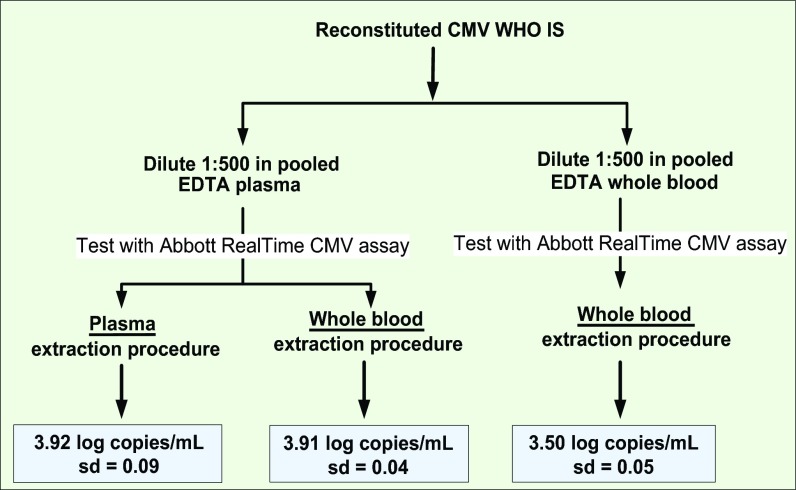
Pilot study dilution and testing scheme to assess CMV WHO IS commutability.

The reconstituted CMV WHO IS was diluted 500-fold in pooled plasma and pooled WB, to a nominal concentration of 4 log IU/ml, and was tested with the Abbott RealTime CMV procedures specific for processing plasma and WB. CMV DNA concentrations were reported in log copies per milliliter. Six replicates were tested per condition. The observed concentrations of the WHO IS diluted in plasma and WB were significantly different (Fig. 1). The measured concentrations of the WHO IS diluted in WB were approximately 0.4 log copies/ml lower than the observed concentration of the WHO IS diluted in plasma: the concentration of the WHO IS diluted in plasma and extracted by the plasma procedure was 3.92 log copies/ml, with a standard deviation (SD) of 0.09, but the concentration was only 3.50 log copies/ml, with an SD of 0.05, when the WHO IS was diluted in WB and extracted by the WB procedure. The difference in viral load was not concentration dependent (for nominal concentrations of 2 to 5 log IU/ml, the differences in quantitation between the WHO IS diluted in plasma and that diluted in WB ranged from 0.40 to 0.51 log copies/ml) (data not shown).

To evaluate the potential contribution from method bias between the two sample processing procedures, plasma dilutions were processed with the WB procedure (Fig. 1). The concentration of the WHO IS was 3.91 log copies/ml, with an SD of 0.04. The observed concentrations of the WHO IS diluted in plasma were comparable regardless of whether sample processing was done with the plasma or the WB procedure, suggesting that the observed lower concentrations of CMV WHO IS dilutions in WB were unlikely to be attributable to a method/procedure bias. It should be noted that WB dilutions can be processed only with the WB procedure, as the plasma sample preparation procedure was not designed to handle the presence of large quantities of background human genomic DNA from WB.

### Evaluation of the matrix effect for the CMV WHO IS.

The pilot observation suggested that quantitation of the CMV WHO IS was affected by the matrix in which the material was diluted. To further assess this potential matrix effect, we tested dilutions of the WHO IS by using patient specimens as the standard of comparison, following the CLSI EP14-A2 guidelines. The reconstituted CMV WHO IS and plasma specimens from CMV-positive patients were diluted in pooled EDTA-plasma and -WB and tested with the Abbott RealTime CMV assay. Three replicates of each patient sample diluted in each matrix and 6 replicates of 3 different concentrations of the WHO IS diluted in each matrix were tested. Concentrations of CMV DNA in the diluted patient samples ranged from approximately 1.4 log copies/ml to 4 log copies/ml. The WHO IS was diluted to nominal concentrations of 3, 3.4, and 4 log IU/ml. Standard deviations ranged from 0.01 to 0.29 log copies/ml (percent coefficient of variation [% CV] = 0.3 to 17.6%) for patient samples and from 0.01 to 0.14 (% CV = 0.18 to 4.28%) for the diluted WHO IS. The % CV was 0.3 to 5% for patient samples with levels above 2 log copies/ml. For patient samples with levels at or below 2 log copies/ml, the % CV ranged from 1.5 to 17.6%. A higher variability at concentrations near the assay LLoQ is characteristic of quantitative PCR assays.

**FIG 2:**
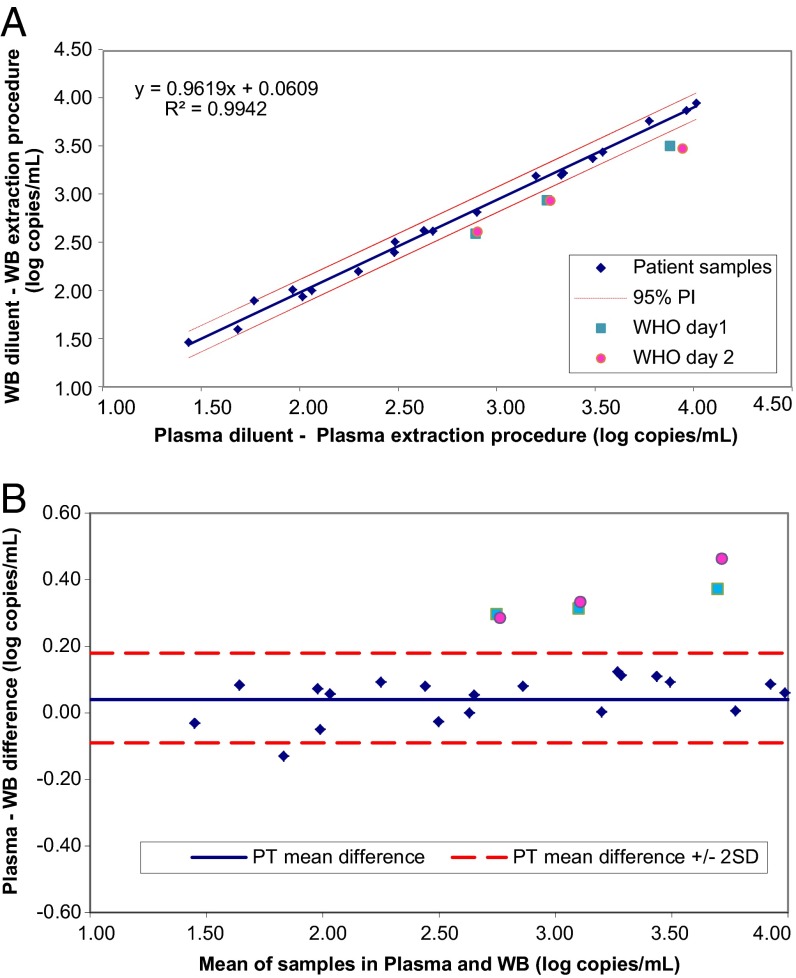
(A) Effect of matrix and extraction method combined on CMV WHO IS quantitation. Patient samples and the WHO IS diluted in plasma and extracted by the plasma procedure (*x* axis) were compared to those diluted in WB and extracted by the WB procedure (*y* axis). This analysis cannot separate the potential effects of the matrix and the extraction procedures. (B) Bland-Altman analysis of the results shown in panel A. The values for mean log copies/ml across samples and the WHO IS diluted in plasma and extracted by the plasma procedure and those diluted in WB and extracted by the WB procedure were plotted against the difference between the two sets of samples. Dashed lines show the mean difference for patient samples (PT) ± 2 SD. 95% PI, 95% prediction interval.

**FIG 3:**
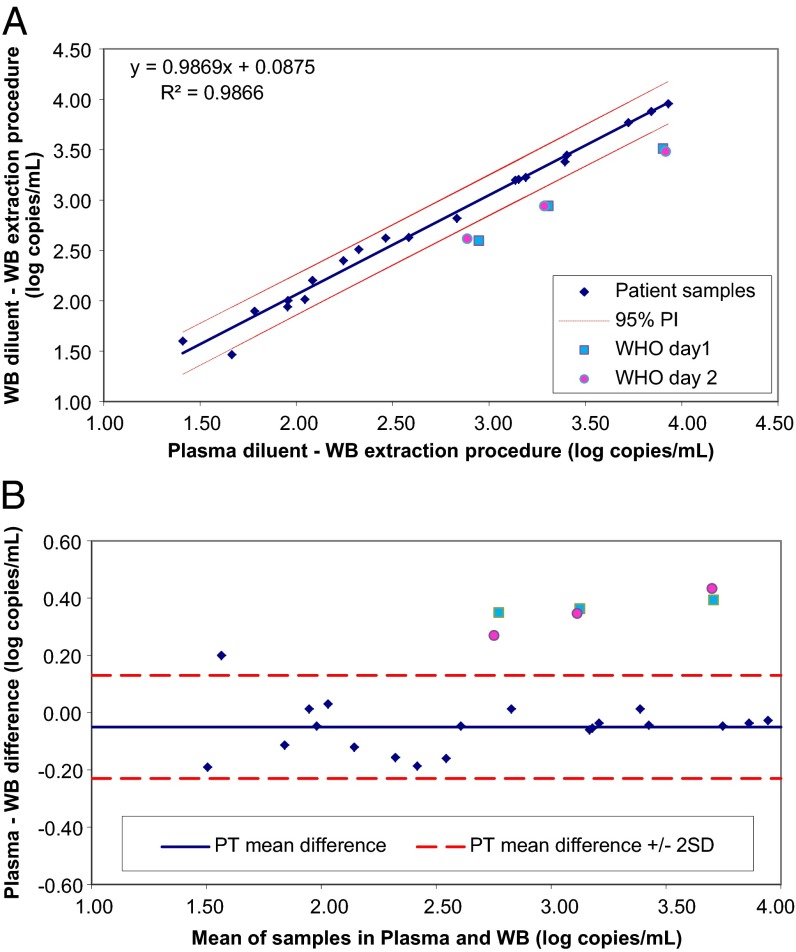
(A) Effect of matrix on WHO IS quantitation. Patient samples and the WHO IS diluted in plasma and extracted by the WB procedure (*x* axis) were compared to those diluted in WB and extracted by the WB procedure (*y* axis). This analysis evaluates the effect of the matrix independently of the extraction procedure. (B) Bland-Altman analysis of the results shown in panel A. The values for mean log copies/ml across samples and the WHO IS diluted in plasma and extracted by the WB procedure and those diluted in WB and extracted by the WB procedure were plotted against the difference between the two sets of samples. Dashed lines show the mean difference for patient samples ± 2 SD.

**FIG 4:**
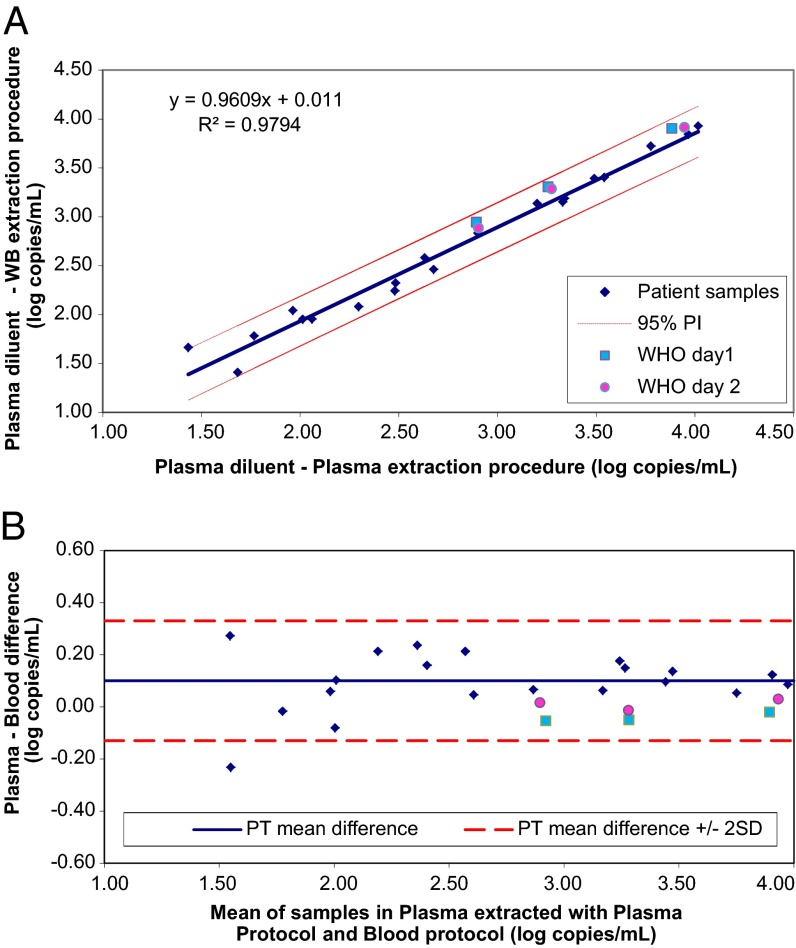
(A) Potential effect of extraction methods on WHO IS quantitation. Patient samples and the WHO IS diluted in plasma and extracted by the plasma procedure (*x* axis) were compared to the same dilutions extracted by the WB procedure (*y* axis). This analysis evaluates the potential effect of the extraction procedure independently of the matrix. (B) Bland-Altman analysis of the results shown in panel A. The values for mean log copies/ml across samples and the WHO IS diluted in plasma and extracted by the plasma procedure and the same dilutions extracted by the WB procedure were plotted against the difference between the two sets of samples. Dashed lines show the mean difference for patient samples ± 2 SD.

Quantitation of CMV DNA from patient samples was plotted following the protocol outlined in CLSI document EP14-A2 (Fig. 2A, 3A, and 4A). The following comparisons were made: dilutions in plasma processed by the plasma extraction procedure versus dilutions in WB processed by the WB extraction procedure (Fig. 2A); dilutions in plasma versus dilutions in WB, both processed by the WB extraction procedure (Fig. 3A); and dilutions in plasma processed by the plasma versus WB extraction procedures (Fig. 4A). For each comparison, a regression line was plotted and the 95% prediction interval for the line was calculated. The slopes were all close to 1, and intercepts were all <0.1 log copies/ml, indicating good agreement for patient samples between test conditions. Results for the CMV WHO IS diluted to nominal concentrations between approximately 3 and 4 log IU/ml were plotted on the same graphs. Values for the CMV WHO IS dilutions that fall outside the 95% prediction interval indicate that the IS behaved differently than the patient samples. Bland-Altman analysis was also used to compare CMV quantitation results obtained with the different matrices and extraction methods (Fig. 2B, 3B, and 4B).

The effect of matrix and extraction method combined on CMV WHO IS quantitation is shown in Fig. 2A and B. In this analysis, the potential effects from the matrix and the extraction procedure cannot be separated. Patient samples and the WHO IS diluted in plasma and extracted by the Abbott RealTime CMV plasma procedure (*x* axis) were compared to those diluted in WB and extracted by the WB procedure (*y* axis) (Fig. 2A). Each point represents the mean for 3 replicates (1 replicate per run for 3 runs). The means for the WHO IS dilutions were plotted relative to the regression line for patient samples and the 95% prediction interval around the line. For each sample, the 3 replicates were tested on the same day, but in order to accommodate the large number of samples, the patient samples were divided into 2 groups and run on 2 days. Three replicates of the WHO IS dilutions were run on both days, and the means for both days are plotted separately (as day 1 and day 2). CMV WHO IS dilutions in plasma and WB were prepared and run on the first test day and then stored frozen at −70°C for 7 days before being thawed for the second run. CMV WHO IS dilutions fell outside the 95% prediction interval, indicating that the WHO IS performed differently from patient samples. Bland-Altman analysis of the results is shown in Fig. 2B. The difference in quantitation between patient samples diluted and extracted under the two sets of test conditions was close to zero. There was a bias of 0.3 to 0.4 log copies/ml for the CMV WHO IS.

The effect of matrix alone on CMV WHO IS quantitation is shown in Fig. 3A and B. Since the same WB extraction procedure was used, this analysis evaluated the effect of the matrix independently of the extraction procedure. Patient samples and the WHO IS diluted in plasma and extracted by the WB procedure (*x* axis) were compared to those diluted in WB and extracted by the WB procedure (*y* axis) (Fig. 3A). CMV WHO IS dilutions from both days fell outside the 95% prediction interval, indicating that the WHO IS performed differently from patient samples and that the effect can be attributed to the dilution matrix. Bland-Altman analysis of the results is shown in Fig. 3B. The difference in quantitation between patient samples was close to zero regardless of whether the diluent was plasma or WB. There was a bias of 0.3 to 0.4 log copies/ml for the CMV WHO IS.

To confirm that the processing procedure did not contribute to the difference between patient samples and the CMV WHO IS, the results from the two extraction procedures were compared (Fig. 4A and B). Patient samples and the CMV WHO IS diluted in plasma and extracted by the plasma procedure (*x* axis) were compared to the same dilutions extracted by the WB procedure (*y* axis) (Fig. 4A). In this case, results for the WHO IS do fall within the 95% prediction interval, indicating that when it is diluted in plasma, the CMV WHO IS is quantitated similarly to patient specimens, regardless of the extraction procedure used. Bland-Altman analysis of the results is shown in Fig. 4B. The difference in quantitation between patient samples was close to zero regardless of whether the plasma extraction procedure or the WB extraction procedure was used. For the CMV WHO IS diluted in plasma, the difference in quantitation was also close to zero, indicating that the two extraction procedures are equally efficient.

### Evaluation of the matrix effect for the NIST SRM.

**FIG 5:**
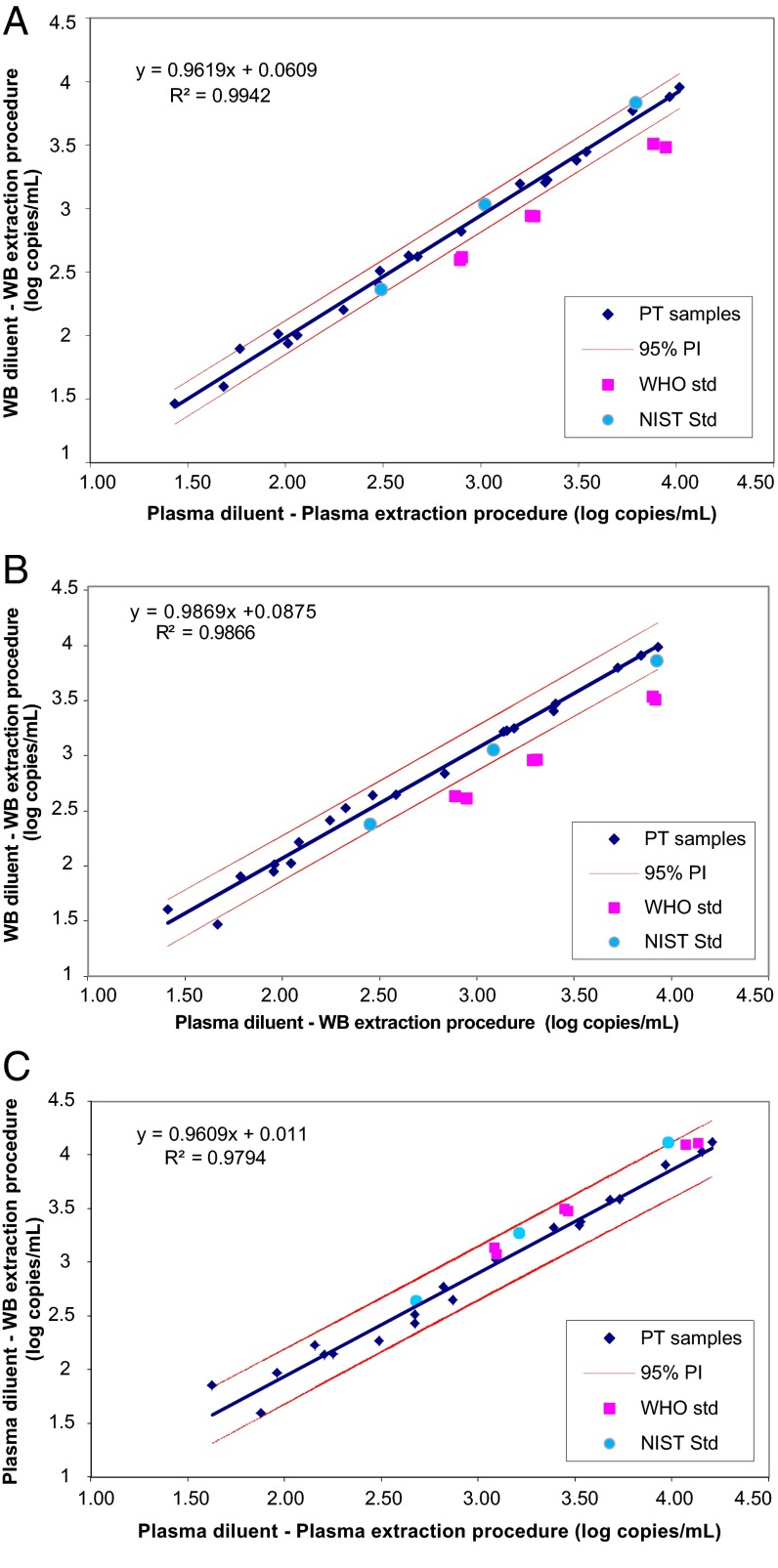
(A) Potential effect of matrix and extraction method on NIST SRM quantitation. Patient samples and the NIST SRM diluted in plasma and extracted by the plasma procedure (*x* axis) were compared to those diluted in WB and extracted by the WB procedure (*y* axis). Results for the WHO IS were also plotted for comparison. (B) Potential effect of matrix on NIST SRM quantitation. Patient samples and the NIST SRM diluted in plasma and extracted by the WB procedure (*x* axis) were compared to those diluted in WB and extracted by the WB procedure (*y* axis). Results for the WHO IS were also plotted for comparison. (C) Potential effect of extraction method on NIST SRM quantitation. Patient samples and the NIST SRM diluted in plasma and extracted by the plasma procedure (*x* axis) were compared to the same dilutions extracted by the WB procedure (*y* axis). Results for the WHO IS were also plotted for comparison.

NIST SRM 2366 was evaluated to determine the potential impact from the matrices used for dilution. NIST SRM components A, B, and C were diluted in pooled EDTA-plasma and -WB, and the quantitation was performed relative to patient samples. The NIST SRM dilutions were not run on the same day as the patient samples, but dilutions of 3 of the patient samples were rerun as controls and fell within the 95% prediction interval defined by the samples in the original runs (data not shown). The means for the NIST standard dilutions were plotted relative to the regression line for patient samples and the 95% prediction interval around the line (Fig. 5A to C). Results for the WHO IS were also plotted for comparison. In contrast to the CMV WHO IS, the NIST SRM fell within the 95% prediction interval for the patient samples, indicating that the NIST SRM behaves similarly to patient samples under the conditions tested.

### CMV WHO IS quantitation in buffer.

Quantitation of the CMV WHO IS diluted in buffer was compared to the results for dilutions in plasma and WB (Table 1). The WHO CMV IS was diluted 1:500, to a nominal concentration of 4 log IU/ml, in each of the diluents. Samples were processed by the WB extraction procedure. Quantitation of the WHO IS diluted in TE buffer was similar to that for dilution in plasma. The value for the WHO IS diluted in WB was lower.

**TABLE 1:** Comparison of CMV WHO IS quantitation results for plasma, WB, and buffer^*a*^

Parameter	Value for diluent		
	Buffer	Plasma	WB
Mean log copies/ml	3.94	3.85	3.57
SD	0.08	0.05	0.04
% CV	2.1	1.2	1.2
*n*	6	6	6

a The reconstituted CMV WHO IS was diluted 1:500, to a nominal concentration of 4.00 log IU/ml. Samples were extracted by the WB procedure.

### NIST SRM quantitation in buffer.

Quantitation of the NIST SRM diluted in buffer was compared to that for the material diluted in plasma and WB (Table 2). NIST SRM component C was diluted 1:2,000, to a nominal concentration of 3.99 log copies/ml, in each of the diluents. Samples were processed by the WB extraction procedure. Quantitation of the NIST standard diluted in buffer was the same as that for the standard diluted in plasma and WB.

**TABLE 2:** Comparison of NIST SRM quantitation results for plasma and buffer^*a*^

Parameter	Value for diluent		
	Buffer	Plasma	WB
Mean log copies/ml^*b*^	3.78	3.72	3.64
SD	0.06	0.06	0.05
% CV	1.7	1.7	1.4
*n*	6	6	6

a NIST SRM component C was diluted 1:2,000, to a nominal concentration of 3.99 log copies/ml. Samples were extracted by the WB procedure.

b Determined per the instructions of the Abbott RealTime CMV assay and standardized to internally prepared DNA material.

It should be noted that DNA copy number assignment for component C dilution differed approximately 0.3 log from that generated by the RealTime CMV assay (a nominal concentration of 3.99 log copies/ml versus a reported concentration of 3.64 log copies/ml). This likely reflects the difference in DNA copy number assignment between the SRM material, which was quantitated by digital PCR, and the dual plasmid used to calibrate the RealTime CMV assay, which was quantitated by the absorbance at 260 nm.

## DISCUSSION

A goal of laboratory medicine is that results for patient samples be comparable, independently of the medical laboratories that produce the results. Commutability of reference materials is critical to the ability to unify results among different methods. Calibration of testing methods with reference materials that are not commutable can cause poorer rather than improved agreement of results for clinical samples (9, 15). A number of methods are available to assess the commutability of a reference material (8, 16).

We evaluated the commutability of the 1st CMV WHO IS by using two measurement procedures of the Abbott RealTime CMV assay. Twenty CMV-positive patient plasma samples were used as the standard of comparison. Potential effects from diluent matrices and measurement procedures were assessed. Patient samples produced similar CMV DNA quantitation results regardless of the diluent or measurement procedure used, indicating that there is no matrix effect associated with plasma or WB diluent used for patient samples and that there is no method bias between the two measurement procedures. The CMV WHO IS, on the other hand, was not commutable with patient specimens and exhibited a matrix effect when diluted in WB. This matrix effect reduced the apparent DNA quantitation of the CMV IS by approximately 0.4 log copies/ml, or 2.5-fold. Calibrating the assay by using the CMV WHO IS diluted in whole blood would introduce a bias for CMV whole-blood quantitative testing; samples would be reported to have higher measured concentrations, by approximately 0.4 log IU/ml. The reason for the matrix-dependent quantitation difference is unknown. It is not due to a bias between the WB and plasma methods, as the quantitation difference was seen when IS samples diluted in WB and plasma were tested side by side with the WB method. It is also not likely to be due to interference from high levels of background genomic DNA in the WB diluent, because the IS levels tested were well within the linear range of the assay for WB specimens. While viral load changes within 0.5 log IU/ml are considered insignificant in the management of an individual patient, the matrix effect examined here has the potential to introduce a systematic bias between labs that standardize relative to the WHO IS by using WB versus plasma as the matrix. A bias of this type poses potential challenges in the consistency of unifying results from different methods and in establishing uniform treatment guidelines based on viral load.

The CMV WHO IS diluted in plasma was commutable relative to patient samples diluted in plasma for the both the plasma and WB procedures (Fig. 4A). Since patient samples gave comparable results when diluted in either plasma or whole blood (Fig. 2B and 3B), the WHO IS diluted in plasma is commutable with patient samples diluted in either plasma or WB. To eliminate the bias observed when the WHO IS was diluted in WB, the WHO IS was diluted in plasma to determine a conversion factor for standardization of the RealTime CMV assay. The same conversion factor was applied to both the plasma and WB procedures, based on the commutability of the WHO IS diluted in plasma.

The observations reported here may explain the apparent discrepancy between the copy number-to-IU conversion factors for the RealTime CMV assay (+0.19 log to convert from log copies/ml to log IU/ml) and higher conversion factors calculated using the CMV WHO IS diluted in WB in recent publications (+0.45 log to convert from log copies/ml to log IU/ml and ×7.69 to convert from copies/ml to IU/ml, which is equivalent to 0.89 log to convert from log copies/ml to log IU/ml) (17, 18). A different conversion factor was also calculated for the RealTime CMV assay by using plasma as a diluent (0.36 log for converting from log copies/ml to log IU/ml) (19). This difference may be attributable to the methods used to determine the factor.

The identification of a matrix effect with the CMV WHO IS underscores the importance of assessing the commutability of the IS in different matrices in order to achieve consistent standardization across different detection methods. Given the matrix effect identified for the reconstituted CMV WHO IS, revision to the instructions of use should be considered. The revision should caution users on the potential impact of the diluent matrix and suggest characterization in order to define an appropriate matrix. For laboratories conducting routine testing of whole-blood CMV specimens, plasma may be an appropriate matrix for dilution of the CMV WHO IS. It is worth noting that the quantitation results for the CMV WHO IS were found to be comparable when the IS was diluted in plasma or buffer. An alternative, easily obtainable diluent matrix should be a possible consideration for the CMV WHO IS.

Commutability is a method-specific characteristic; a reference material may be commutable for some measurement procedures but noncommutable for others (8, 16). A recent study compared the commutabilities of the CMV WHO IS diluted in plasma across 10 quantitative real-time PCR assays (16). Assays were compared pairwise by linear regression to assess the matrix effect and as larger groups by using correspondence analysis. The degree of commutability of the CMV WHO IS varied depending on which test methods were compared and on the method used to analyze the data (16). The “fitness for use” of a reference material is dependent on the measurement procedures for which it is found to be commutable.

Although the impact of noncommutable reference materials is well documented and international standards and guidance documents require reference materials to be validated for commutability (10, 11), the assessment of commutability of reference materials is not routinely performed (8). There are considerable logistical difficulties in designing and executing a multisite commutability study. Further, the matrix effect described here and the variable commutability between test methods (16) highlight additional levels of complexity for such studies.

There have been substantial recent efforts to evaluate the commutability of secondary, commercially available CMV calibration materials (20, 21). These studies have identified calibration materials that are commutable between some laboratory methods but noncommutable for other laboratory method pairs (20). Because of the difficulties in sourcing adequate quantities of patient-based CMV DNA-positive materials, both the CMV WHO IS and the available secondary commercial calibration materials are CMVs propagated in tissue culture. A CMV AD169 strain preparation purchased as a commercial calibration material was identified by our measurement procedure as being associated with a matrix effect in whole blood, similar to that for the CMV WHO IS (data not shown). Since strain AD169 (and other tissue culture CMVs) is frequently used for the preparation of panels for external quality assessment (proficiency testing), it is critical to further evaluate the commutability of both primary and secondary CMV reference materials, including those used in external quality assessments across different quantitative molecular diagnostic methods and for both plasma and whole blood matrices. Without commutability assessment, a measurement bias uncovered by proficiency testing cannot be attributed properly to the measurement procedure or calibration, and the acceptability of the performance of measurement procedures cannot properly be determined (8).

The standard reference material NIST SRM 2366, made of purified CMV DNA, became available in late 2011. The material was quantified via digital PCR and was assigned a concentration given in DNA copies per microliter. NIST SRM 2366 was tested using the two measurement procedures of the RealTime CMV assay. Although previous testing of the NIST SRM involved no upfront sample preparation (6), the material was diluted in plasma and WB in our study and was processed through the entire measurement procedures, along with patient samples. The material was found to be commutable with patient samples. In addition, quantitation results for the material were found to be comparable when it was diluted in plasma or buffer. As suggested previously, the establishment of this independently produced reference material may have significant value when evaluated in conjunction with the international biological standard (22). The development of the CMV WHO IS brings significant benefits to the field. The matrix effect and the variable commutability between test methods highlight the complexity of developing a truly commutable standard for CMV and point to the need to better understand the CMV WHO IS. It should be noted that the impact of a matrix effect and variable commutability on CMV quantitation may not be limited to the CMV WHO IS. Discordant results may be observed with any standard, panel, or secondary calibrator. Quantitation of standards, panels, and secondary calibrators must be interpreted with caution in light of these findings.

## References

[1] Kotton CN, Kumar D, Caliendo AM, Asberg A, Chou S, Danziger-Isakov L, Humar A 2013 Updated international consensus guidelines on the management of cytomegalovirus in solid organ transplantation. Transplantation 96:333–360. doi:10.1097/TP.0b013e31829df29d. 23896556

[2] Dykewicz CA 2001 Summary of the guidelines for preventing opportunistic infections among hematopoietic stem cell transplant recipients. Clin Infect Dis 33:139–144. doi:10.1086/321805. 11418871

[3] Andrews PA, Emery VC, Newstead C 2011 Summary of the British transplantation society guidelines for the prevention and management of CMV disease after solid organ transplantation. Transplantation 92:1181–1187. doi:10.1097/TP.0b013e318235c7fc. 22002346

[4] Avery RK 2007 Management of late, recurrent, and resistant cytomegalovirus in transplant patients. Transplant Rev 21:65–76. doi:10.1016/j.trre.2007.02.001.

[5] Fryer JF, Heath AB, Anderson R, Minor PD, Collaborative Study Group. 2010 Collaborative study to evaluate the proposed 1st WHO International Standard for Human Cytomegalovirus for Nucleic Acid Amplification-Based Assays. WHO/BS/10.2138. WHO Expert Committee on Biological Standardization, Geneva, Switzerland.

[6] Haynes RJ, Kline MC, Toman B, Scott C, Wallace P, Butler JM, Holden MJ 2013 Standard reference material 2366 for measurement of human cytomegalovirus DNA. J Mol Diagn 15:177–185. doi:10.1016/j.jmoldx.2012.09.007. 23321018

[7] Fasce CF, Rej R, Copeland WH, Vanderlinde RE 1973 A discussion of enzyme reference materials: applications and specifications. Clin Chem 19:5–9. 4683368

[8] Vesper HW, Miller WG, Myers GL 2007 Reference materials and commutability. Clin Biochem Rev 28:139–147. 18392124 PMC2282402

[9] Miller WG, Myers GL, Rej R 2006 Why commutability matters. Clin Chem 52:553–554. doi:10.1373/clinchem.2005.063511. 16595820

[10] International Organization for Standardization (ISO). 2002 In vitro diagnostic systems—measurement of quantities in samples of biological origin—description of reference materials. ISO 15194:2002. ISO, Geneva, Switzerland.

[11] International Organization for Standardization (ISO). 2003 In vitro diagnostic medical devices—measurement of quantities in biological samples—metrological traceability of values assigned to calibrators and control materials. ISO 17511:2003. ISO, Geneva, Switzerland.

[12] Clinical and Laboratory Standards Institute (CLSI). 2005 Evaluation of matrix effects; approved guideline, 2nd ed CLSI document EP14-A2. Clinical and Laboratory Standards Institute, Wayne, PA.

[13] Bland JM, Altman DG 1986 Statistical methods for assessing agreement between two methods of clinical measurement. Lancet 8:307–310. 2868172

[14] Bland JM, Altman DG 1995 Comparing methods of measurement: why plotting difference against standard method is misleading. Lancet 346:1085–1087. doi:10.1016/S0140-6736(95)91748-9. 7564793

[15] Pang XL, Fox JD, Fentona JM, Millere GG, Caliendo AM, Preiksaitisa JK 2009 Interlaboratory comparison of cytomegalovirus viral load assays. Am J Transplant 9:258–268. doi:10.1111/j.1600-6143.2008.02513.x. 19178413

[16] Hayden RT, Preiksaitis J, Tong Y, Pang X, Sun Y, Tang L, Cook L, Pounds S, Fryer J, Caliendo AM 2015 Commutability of the First World Health Organization International Standard for Human Cytomegalovirus. J Clin Microbiol 53:3325–3333. doi:10.1128/JCM.01495-15. 26269622 PMC4572535

[17] Schnepf N, Scieux C, Resche-Riggon M, Feghoul L, Xhaard A, Gallien S, Molina J-M, Socié G, Simon F, Mazeron M-C, LeGoff J 2013 Fully automated quantification of cytomegalovirus (CMV) in whole blood with the new sensitive Abbott RealTime CMV assay in the era of the CMV international standard. J Clin Microbiol 51:2096–2102. doi:10.1128/JCM.00067-13. 23616450 PMC3697667

[18] Furione M, Rognoni N, Cabano E, Baldanti F 2012 Kinetics of human cytomegalovirus (HCMV) DNAemia in transplanted patients expressed in international units as determined with the Abbott RealTime CMV assay and an in-house assay. J Clin Virol 55:317–322. doi:10.1016/j.jcv.2012.08.017. 22989927

[19] Clari MÁ, Bravo D, Costa E, Muñoz-Cobo B, Solano C, Remigia MJ, Giménez E, Benmarzouk-Hidalgo OJ, Pérez-Romero P, Navarro D 2013 Comparison of the new Abbott Real Time CMV assay and the Abbott CMVPCR kit for the quantitation of plasma cytomegalovirus DNAemia. Diagn Microbiol Infect Dis 75:207–209. doi:10.1016/j.diagmicrobio.2012.10.010. 23182073

[20] Caliendo AM, Shahbazian MD, Schaper C, Ingersoll J, Abdul-Ali D, Boonyaratanakornkit J, Pang XL, Fox J, Preiksaitis J, Schönbrunner ER 2009 A commutable cytomegalovirus calibrator is required to improve the agreement of viral load values between laboratories. Clin Chem 55:1701–1710. doi:10.1373/clinchem.2009.124743. 19574467

[21] Hayden RT, Shahbazian MD, Valsamakis A, Boonyaratanakornkit J, Cook L, Pang XL, Preiksaitis JK, Schönbrunner ER, Caliendo AM 2013 Multicenter evaluation of a commercial cytomegalovirus quantitative standard: effects of commutability on interlaboratory concordance. J Clin Microbiol 51:3811–3817. doi:10.1128/JCM.02036-13. 24025907 PMC3889771

[22] Madej RM, Davis J, Holden MJ, Kwang S, Labourier E, Schneider GJ 2010 International standards and reference materials for quantitative molecular infectious disease testing. J Mol Diagn 12:133–143. doi:10.2353/jmoldx.2010.090067. 20075208 PMC2871718

